# Whole-Genome Sequences of Staphylococcus pseudintermedius Isolates from Canine and Human Bacteremia Infections

**DOI:** 10.1128/MRA.00735-19

**Published:** 2019-07-11

**Authors:** Sara V. Little, Laura K. Bryan, Andrew E. Hillhouse, Kranti Konganti, Sara D. Lawhon

**Affiliations:** a Department of Veterinary Pathobiology, College of Veterinary Medicine & Biomedical Sciences, Texas A&M University, College Station, Texas, USA; b Texas A&M Institute for Genome Sciences and Society, College of Veterinary Medicine & Biomedical Sciences, Texas A&M University, College Station, Texas, USA; University of Rochester School of Medicine and Dentistry

## Abstract

Here, we report the complete and draft genome sequences of 8 Staphylococcus pseudintermedius isolates, 4 from human bacteremia infections and 4 from canine bacteremia infections. This species is recognized primarily as an important canine pathogen, but it is increasingly being identified in human infections.

## ANNOUNCEMENT

Staphylococcus pseudintermedius is a major canine pathogen that, similarly to Staphylococcus aureus, has an increasing trend of multiple-drug resistance. The identification of S. pseudintermedius as a human pathogen has increased (1–6). Isolates may be misidentified as S. aureus, which can complicate treatment (5, 7, 8). The ability of staphylococci to exit the bloodstream and establish secondary infections in a myriad of tissues suggests a broad arsenal of virulence factors (9–11). Whole-genome sequencing of isolates from both humans and dogs may provide valuable insights into the virulence factors of the organism, as well as into potential differences in isolates causing human infections. Here, we present 8 whole-genome sequences of S. pseudintermedius from human and canine bacteremia cases.

Four human bacteremia isolates were acquired from a collection of 45 human isolates. A description of the isolates and their collection was previously published (5). Four canine bacteremia isolates were selected from isolates collected by the Clinical Microbiology Laboratory of the Texas Veterinary Medical Teaching Hospital at Texas A&M University between 2007 and 2016. Isolates were stored in lysogeny broth supplemented with 10% glycerol at −80°C at the time of isolation. Isolates were originally identified, as previously described, as S. pseudintermedius, and identification was confirmed using multiplex PCR and matrix-assisted laser desorption ionization–time of flight (MALDI-TOF) mass spectrometry (5, 12). After sequencing, multilocus sequence typing (MLST) and ribosomal multilocus sequence typing (rMLST) were used to further confirm identification as S. pseudintermedius (13, 14). Prior to sequencing, isolates were revived by growing them on blood agar for 24 hours at 37°C. Isolates were subcultured twice to ensure purity. A single colony was inoculated into Bacto brain heart infusion broth (BHIB; Becton, Dickinson and Company, Franklin Lakes, NJ, USA) and grown for 8 hours at 37°C prior to extraction.

For Illumina sequencing, 1-ml aliquots of each isolate in BHIB were pelleted and lysed in a Qiagen TissueLyser using Macherey-Nagel bead tubes type B and lysis buffer from the NucleoMag tissue DNA kit. DNA isolation followed the manufacturer’s protocol (Macherey-Nagel). Libraries were prepared using the Illumina Nextera DNA Flex library preparation kit following the manufacturer’s protocol and sequenced with an Illumina MiSeq V2 2 × 250-bp kit. All data were uploaded to Illumina’s cloud-based resource, BaseSpace, for run monitoring, FASTQ generation, demultiplexing, and adapter trimming. The sequencing output of paired-end read sets contained approximately 1.5 million reads/isolate of 250 bp, resulting in approximately 300× coverage.

For MinION sequencing, DNA was extracted from a 1-ml aliquot of each isolate in BHIB using a MasterPure Gram-positive DNA purification kit per the standard protocol, with the addition of lysostaphin during the lysing incubation. Libraries were prepared following the manufacturer’s protocol for 1D PCR barcoding of genomic DNA using the Nanopore SQK-LSK108 kit. Genomic DNA quality was verified on a genomic DNA TapeStation run (Agilent). Data collection was performed by MinKNOW software, utilizing the following workflow: NC_48Hr_Sequencing_Run_FLO_MIN106_SQK-LSK108.py. MinION sequencing data were demultiplexed and base called using Albacore Sequencing Pipeline Software version 1.2.4. Sequencing resulted in an output of read sets containing 100 to 500 Mb per sample, resulting in 40× to 200× coverage.

MinION reads were assembled using Canu version 1.5 (15). The draft genomes were polished using Pilon version 1.22 (16); the initial polishing run utilized BAM files of MiSeq reads mapped to the Canu draft genome (Bowtie2 version 2.3.4.3) (17, 18), and the subsequent runs used MiSeq reads mapped to the previously polished draft genome for a total of 3 polishing runs. The genomes were analyzed for completeness using Benchmarking Universal Single-Copy Orthologs (BUSCO) version 3 (19, 20) using the *Firmicutes* OrthoDB version 9 data set. Genomes were scaffolded using MeDuSa version 1.6 (21), using 3 reference genomes, namely, ED99 (GenBank accession number CP002478) and the two single-contig assemblies from this study (Tamu 51_92 and Tamu 53_60). Default parameters were used for all software unless otherwise specified.

The final assemblies resulted in genomes between 2,561,987 bp and 2,615,859 bp (Table 1). The genomes consisted of 1 to 29 contigs per isolate, with the largest contigs ranging from 2,615,859 to 420,164 bp. Two genomes were complete single-contig assemblies, 2 were single-scaffold assemblies, and 4 were draft genomes of 2 to 3 scaffolds. BUSCO scores for the genomes ranged from 98.7% to 99.6%.

**TABLE 1:** Characteristics and accession numbers of genome sequences of S. pseudintermedius bacteremia isolates^*a*^

Isolate	Source	No. of contigs	No. of scaffolds	BUSCO (%)	*N*_50_ (bp)	Genome size (bp)	G+C content (%)	GenBank accession no.	SRA accession no.		
									MinION FastQ	MinION Fast5	MiSeq
Tamu 46_57	Canine	13	2	99.20	638,705	2,561,987	37.7	SEZZ00000000	SRR8538958	SRR9211302	SRR8538959
Tamu 49_44	Canine	20	1	98.70	385,638	2,583,863	37.67	CP035743	SRR8538960	SRR9211301	SRR8538961
Tamu 50_21	Canine	7	2	99.60	1,401,107	2,527,337	37.74	SEZY00000000	SRR8538954	SRR9211304	SRR8538955
Tamu 51_92	Canine	1	1	99.60	2,512,263	2,512,263	37.8	CP035742	SRR8538956	SRR9211303	SRR8538957
Tamu 53_58	Human	24	2	99.60	238,709	2,655,352	37.42	SEZX00000000	SRR8538952	SRR9211298	SRR8538953
Tamu 53_60	Human	1	1	99.60	2,615,859	2,615,859	37.47	CP035741	SRR8538946	SRR9211297	SRR8538947
Tamu 53_63	Human	29	3	99.60	191,852	2,628,670	37.59	SEZW00000000	SRR8538948	SRR9211300	SRR8538949
Tamu 53_88	Human	5	1	99.60	1,396,228	2,593,641	37.69	CP035740	SRR8538950	SRR9211299	SRR8538951

a BioProject accession number PRJNA521119.

### Data availability.

This whole-genome project has been deposited in DDBJ/ENA/GenBank under the accession number PRJNA521119. SRA and genome accession numbers are listed in Table 1. This announcement presents the first version of each genome.
